# Study of the Counter Anions in the Host-Guest Chemistry of Cucurbit[8]uril and 1-Ethyl-1′-benzyl-4,4′-bipyridinium

**DOI:** 10.1155/2013/452056

**Published:** 2013-05-27

**Authors:** Hailong Ji, Fengyu Liu, Shiguo Sun

**Affiliations:** College of Science, Northwest A&F University, Yangling, Shaanxi 712100, China

## Abstract

A series of 1-ethyl-1′-benzyl-4,4′-bipyridinium compounds with different counter anions (BEV-X_2_, where the X is Cl, Br, I, PF_6_, ClO_4_) were synthesized. By using of NMR, MS, electrochemistry, Na_2_S_2_O_4_-induced redox chemistry, and UV-Vis, the role of the different counter anions in the host-guest chemistry of cucurbit[8]uril (CB[8]) was studied for the first time. The result demonstrated that BEV-X_2_ can form a 1 : 1 host-guest complex with CB[8] in water. Theoretical calculation further suggested that the viologen region was threaded through the cavity of CB[8], while the corresponding counter anions were located outside the cavity. Some difference can be observed on UV-Vis titration and Na_2_S_2_O_4_-induced redox chemistry, which showed that the counter anions have some effect on the host-guest chemistry. All these provide new insights into CB[8] host-guest system.

## 1. Introduction

Since Freeman [1] and coworkers revealed its barrel-shaped structure for the first time in 1981, cucurbit[*n*]uril (CB[*n*], *n* = 5–12) have received great attentions in many areas. Among them, CB[8] is the most interesting because it can accommodate two identical [2–5] or different [6–12] aromatic guests in its cavity. And these kinds of host-guest systems have been employed for the assembly of molecular amphiphiles [6], molecular loops [13, 14], photochemistry reactions control [15, 16], and supramolecular polymers [11, 17]. In the CB[8] host-guest chemistry, viologen derivatives were often used as guest molecules because of their outstanding electron-accepting properties. The inclusion of the methyl viologen (N,N-dimethyl-4,4-bipyridinium, MV^2+^) and derivatives in CB[8] has been studied extensively both by ourselves [18–26] and others [2–14] in recent years, and the results show that CB[8] can bind with MV^2+^ and its radical MV^+•^ strongly. In particularly, CB[8] can form a 2 : 1 including complex with the radical to form a dimer (MV^+•^)_2_/CB[8]; the dimerization constant of MV^+•^ in the presence of CB[8] is estimated to be 2 × 10^7^ M^−1^, which is about 10^5^ times larger than that of MV^+•^ alone in aqueous media. The characteristic UV-Vis absorption peaks for the radical dimer are around 370 nm, 540 nm, and 900 nm, while those for the free radical are around 400 nm and 600 nm; all these can be employed to distinguish them from each other [2–14, 18–20]. 

As we know, viologen derivatives have two positive charges in the structure, and two counter anions are needed to keep the molecule neutral [3, 18–26]. Although the host-guest chemistry of viologen guest molecules with CB[8] has been studied widely, to the best of our knowledge, no attempt has been made to examine the effect of the counter anion in the CB[8] host-guest system. It should be noted that the counter anions could play crucial roles in supermolecular assembly process of viologen derivatives with the other hosts. For instance, complexation of crown ether derivatives with viologen dicationic guests having univalent counterions included two modes: (1) dissociation of the ion pair prior to interaction of the free dication with the host to produce a complex that is not ion-paired and (2) direct complexation of the ion-pair to produce an ion paired complex [27]. In the formation of a calix[6]arene wheel complex, the tosylate or hexafluorophosphate salts of the bipyridinium-based threads affected both the stability of the complexes and the rate of the threading process [28]. Saielli et al. [29, 30] reported the aggregation behavior of the octyl viologen salt with the hydrophobic anion bis(trifluoromethanesulfonyl)amide in nonpolar solvents such as toluene, benzene, and chloroform. And the associated counter anions were found to be responsible for the formation of the large aggregates and the high solubility of the octyl viologen salt in nonpolar solvents. When two viologen fragments were connected with a propyl linker, the molecule exhibited a higher tendency for a chelate-like complexation with chloride anions in polar DMSO/H_2_O media, where chlorides were found to be complexed through a dense hydrogen bond network [31], while the iodides were found to be driven by charge-transfer processes with the electron-deficient pyridinium rings in the crystal lattice [31]. Furthermore, halide anions were selectively recognized by crosslinked polyviologen film because of different sizes [32], revealing the viologen's potential for anion recognition and transportation. 

**Figure 1:**
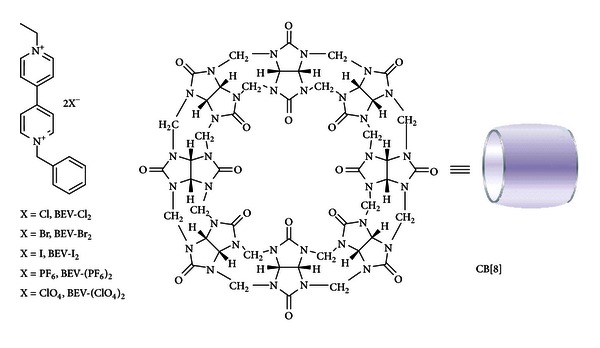
Structure of BEV-X_2_ and CB[8].

 In the previous study [26], 1-ethyl-1′-benzyl-4,4′-bipyridinium dichloride (BEV-Cl_2_) and CB[8] are found to form inclusion complex. It is assumed that the effective positive charge character on the bipyridinium fragment could be affected by the associated counter anions, which could in turn influence the efficiency of CB encapsulation. In an effort to gain further understanding on the CB encapsulation, we have now synthesized a series of 1-ethyl-1′-benzyl-4,4′-bipyridinium compounds that bear different counter anions (BEV-X_2_, Figure 1, where the X is Cl, Br, I, PF_6_, ClO_4_) and reported their inclusion complex behaviour with CB[8].

During the study, the interaction between BEV-Cl_2_ and CB[8] was used as a reference. For comparison, different counter anions were chosen, which included different halides, as well as PF_6_^−^ and ClO_4_^−^. To eliminate the possible interference from the other ions, double distilled water was employed for all the measurement except electrochemistry, where 0.1 M phosphate buffer solutions (pH 7.4) were used instead. The roles of the different counter anions on the CB[8] host-guest chemistry were examined by NMR, MS, electrochemistry, and Na_2_S_2_O_4_-induced redox chemistry.

## 2. Experimental Section

### 2.1. Chemicals and Reagents

All the solvents were of analytic grade, and CB[8] was synthesized according to [33]. The NMR data were recorded on a Varian Inova-400 spectrometer with chemical shifts reported as ppm, and the solvents were D_2_O. Mass spectrometric data were obtained on a Q-Tof mass spectrometer (Micromass, Manchester, UK). Absorption spectra were measured on a Perkin Elmer Lambda 35 UV-Vis spectrophotometer.

Electrochemical measurements were recorded with a BAS 100 B/W electrochemical work station; the scan rate is 10 mV s^−1^ for all cyclic voltammetric (CV) and differential pulse voltammetric (DPV) experiments. All curves were obtained in a three-electrode cell under N_2_. The working electrode was a glassy carbon disc (diameter 3 mm) that was successively polished with 3 *μ*m and 1 *μ*m diamond pastes. The experimental counter electrode was a platinum wire and the reference electrode was a saturated calomel electrode (SCE). The experiments were conducted in 0.1 M phosphate buffer solutions (pH 7.4). All solutions were deoxygenated by purging with N_2_ and maintained under an inert atmosphere during the electrochemical experiments.

### 2.2. Synthetic Section

#### 2.2.1. Syntheses of BEV-Cl_2_, BEV-Br_2_, and BEV-I_2_

BEV-(PF_6_)_2_ was synthesized according to the literature [24]. And the anions were exchanged to the corresponding halide anions by ion exchange experiments. Take BEV-Cl_2_ as an example: BEV-(PF_6_)_2_ (0.56 g, 1 mmol) was dissolved in 3 mL anhydrous acetone and dropped into the solution of tetrabutylammonium chloride (0.6 g, 2 mmol). The precipitate was collected and dried to give BEV-Cl_2_. The corresponding BEV-Br_2_ and BEV-I_2_ were obtained by the same procedure.

#### 2.2.2. Syntheses of BEV-(ClO_4_)_2_

BEV-I_2_ (0.53 g, 1 mmol) was dissolved in 10 mL deionized water and dropped into an aqueous solution of AgClO_4_ (0.41 g, 2 mmol). The precipitate was washed thoroughly with acetonitrile. Then the solvent was removed to get the crude product, which was further purified by recrystallization from acetonitrile.

Not much difference can be observed on ^1^H NMR spectra for BEV-X_2_ with different counter anions in D_2_O (see Figure S1 in Supplementary Material available online at http://dx.doi.org/10.1155/2013/452056); take BEV-Cl_2_ as an example: ^1^H NMR (400 MHz, D_2_O) *δ* 9.14 (dd, *J* = 14.7, 6.9 Hz, 4H), 8.53 (t, *J* = 5.6 Hz, 4H), 7.54 (s, 5H), 5.94 (s, 2H), 4.78–4.73 (m, 2H), and 1.70 (t, *J* = 7.3 Hz, 3H). 

### 2.3. Theoretical Calculation Method

Density functional theory (DFT) calculations were carried out with the Gaussian 09 program package [34], utilizing Becke's three-parameter B3LYP exchange correlation functional together with the 3–21 G basis set for C, H, O, and N atoms [35]. The geometries of the singlet ground state (S_0_) were fully optimized. No imaginary frequencies were computed in the frequency analysis of any of the calculated structures, demonstrating that each is in a local energy minimum.

## 3. Results and Discussion

### 3.1. The Host-Guest Complexes Studies

**Figure 2:**
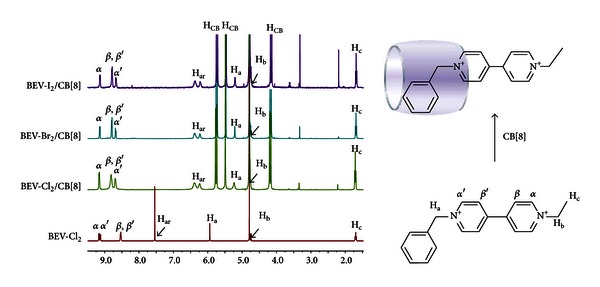
From bottom to up, ^1^H NMR spectra for BEV-Cl_2_ alone, 1 : 1 including complexes of BEV-Cl_2_/CB[8], BEV-Br_2_/CB[8] and BEV-I_2_/CB[8], respectively, and the schematic representation of the assembly of BEV^2+^/CB[8].

As shown in Figure 2, ^1^H NMR spectra of BEV-X_2_ (where the X is Br, I) before and after interaction with CB[8] can be easily followed in D_2_O. It is no doubt that the assembly happened after addition of 1 equivalent CB[8] into the solution of BEV-X_2_, which is in accordance with our previous study [26] on BEV-Cl_2_ and CB[8]. Unfortunately, the corresponding ^1^H NMR spectra of BEV-(PF_6_)_2_ and BEV-(ClO_4_)_2_ were not obtained due to the compounds' poor solubility in water.

Not much difference can be observed for BEV-X_2_ (where the X is Cl, Br, I) with different counter anions on ^1^H NMR spectra with and without the presence of CB[8], suggesting that the counter anions could be located just outside the cavity of CB[8], which have little effect on the electronic environment of the including complexes. 

The formation of 1 : 1 inclusion complex was also confirmed by mass spectrometry (see Supplementary Figure S2). The MS spectra gave a positively charged peak at *m/z* 802.5623, which is calculated for (BEV^2+^ + CB[8])/2. The same peaks can also be found in the ESI-MS spectra of BEV-Br_2_ and BEV-I_2_ with the presence of CB[8], providing strong evidence for the formation of the corresponding host-guest complex, demonstrating that the counter anions exhibited little effect on mass spectrometry of the 1 : 1 including complexes. 

### 3.2. Theoretical Calculation

**Figure 3:**
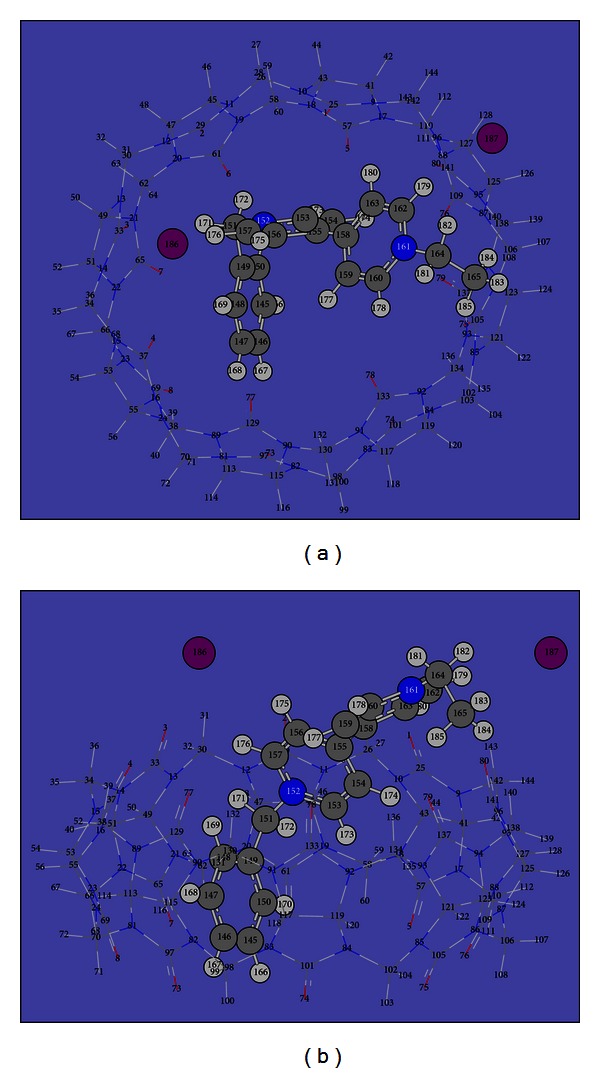
Optimized geometry of the inclusion complex BEV-I_2_/CB[8] viewed from top (a) and front (b). To aid visualization, CB[8] is in stick representation and the viologen salt in ball and stick representation with the iodide atoms in purple.

DFT calculations were carried out to shed some light on the binding. A continuum solvation model (PCM) was used for the consideration of solvent effects in aqueous solution. The optimization result (Figure 3, BEV-I_2_ was taken as an example here; the others can be found in Supplementary Figure S3) showed that the benzyl unit and part of viologen unit were inserted into the CB[8] cavity, while a relatively larger part of viologen was exposed outside the CB[8] cavity. The benzyl unit was bent towards the bipyridinium ring inside the CB[8] cavity, leading to the result that the pyridinium ring was somehow rotated around the central C_155_–C_158_ bond and the two rings were no longer kept on the same plane.

The I^−^ counter anions, depicted in purple, were shown to be located outside the CB[8] cavity. The distances between I^−^ and the nitrogens atom are 5.26 Å and 4.56 Å, respectively, providing further evidence that there is electrostatic interaction between I^−^ and CB[8] host. The distances between I^−^ and the nearest hydrogen atom are 3 Å and 2.79 Å, suggesting that some weak hydrogen bonding interaction might exist.

To make a comparison, the key optimized atom distances of C_146_-N_152_, the angles of A(C_149_-C_151_-N_152_), and the related dihedral angles for the including complexes in the ground states are listed in Table 1.

It is obvious that the BEVI_2_ guest was distorted more severely than the others inside the CB[8] cavity, which might influence the efficiency of CB encapsulation.

### 3.3. The Electrochemical Behavior

The electrochemical properties of all the target complexes were studied in a phosphate buffer solution (0.1 M, pH 7.4), with a scan rate of 10 mV/s both in DPV and CV detection (see Supplementary Figure S4). The concentration of BEV-X_2_ was 0.5 mM. All of the five complexes have nearly the same two consecutive one-electron reduction peaks; the first wave around −0.53 V was attributed to the reduction of EV^2+^ moiety to the cation radical EV^+•^ while the second wave around −0.80 V corresponded to the reduction of EV^+•^ to EV^0^. After addition of 1 equivalent CB[8], the first peak potential was shifted to about −0.55 V while the second peak shifted to about −1.10 V. The reason that the second peak shifted so much can be ascribed to the formation of radical dimer in the cavity of CB[8]. All these are in good agreement with our previous result [23]. Not too much difference caused by different counter anions can be observed during the measurements, providing further evidence that the counter anions did not have much influence on the CB[8] host during electrochemistry. 

 A control experiment was done on the buffer solution alone without addition of any complexes (see Supplementary Figure S5). No peak signals can be observed either in DPV or CV detections, demonstrating that the counter anions are entirely responsible for the above mentioned electrochemical results.

### 3.4. UV-Vis Studies for the Including System

**Figure 4:**
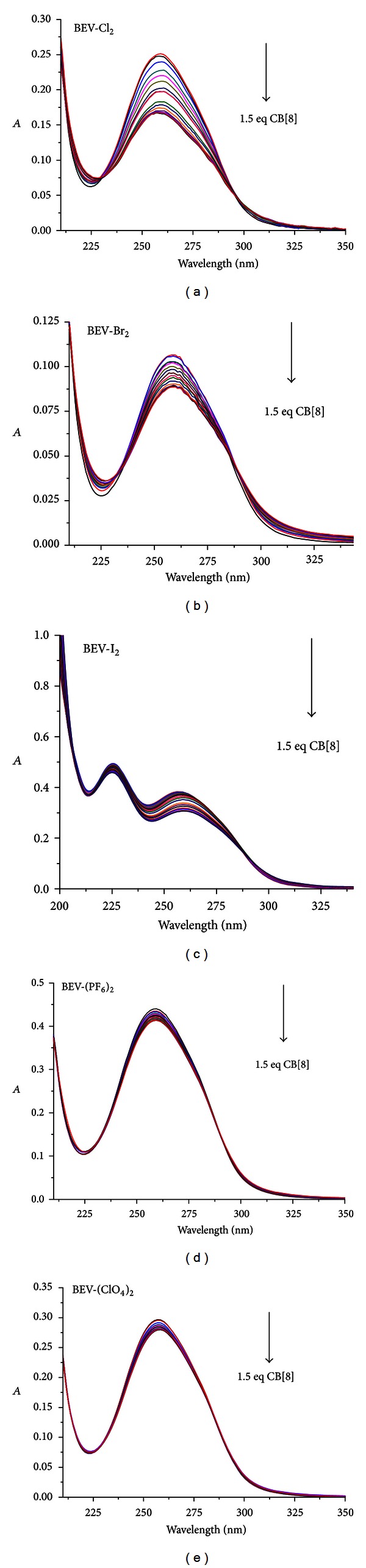
The absorption spectra of BEV-X_2_ (1 × 10^−5^ M) with the addition of different equivalents of CB[8], (a) BEV-Cl_2_, (b) BEV-Br_2_, (c) BEV-I_2_, (d) BEV-(PF_6_)_2_, and (e) BEV-(ClO_4_)_2_, respectively. The concentrations of CB[8] added are 0.05, 0.075, 0.1, 0.125, 0.15, 0.175, 0.2, 0.225, 0.25, 0.275, 0.5, 0.525, 0.75, 1.0, 1.25, and 1.5 equivalents, respectively. The arrows indicate how the absorption bands respond to the increases of the CB[8] concentration.

The interaction of BEV-X_2_ with CB[8] was also studied on UV-Vis in aqueous solution as shown in Figure 4.

 The concentration of BEV-X_2_ was 0.01 mM. All BEV-X_2_ have the same absorption peak at around 260 nm, which belonged to the absorption of MV^2+^ [26]. In the case of BEV-I_2_, another absorption peak centered at 226 nm can be found; this was attributed to the absorption of I^−^ [36]. With the addition of CB[8], the absorption intensity at 260 nm was clearly decreased, suggesting that the assembly process took place. This is in good agreement with the literature [2, 26]. However, the peak at 226 nm showed less reducing effect in comparison with that of 260 nm, demonstrating that the host-guest including process had less effect on the absorption of I^−^; the same effect can be followed in control experiment on UV-Vis titration of 10 *μ*M KI with CB[8] (see Supplementary Figure S6), suggesting that some weak interaction existed between the counter anion I^−^ and CB[8], which is in good agreement with the above mentioned results. According to the literature method [20], the binding constant of BEV-Cl_2_ in the presence of CB[8] is 6.29 × 10^5^ M^−1^ by sigmoidal fitting, while those for BEV-Br_2_, BEV-I_2_, BEV-(PF_6_)_2_, and BEV-(ClO_4_)_2_ are 2.37 × 10^5^ M^−1^, 3.37 × 10^4^ M^−1^, 1.92 × 10^5^ M^−1^ and 5.03 × 10^5^ M^−1^, respectively (see Supplementary Figure S7). The lower binding constant of BEV-I_2_ can be ascribed to the afore mentioned weak interaction between I^−^ and CB[8], and the more twisted conformation of BEV inside CB[8]; the real reason is under investigation.

**Figure 5:**
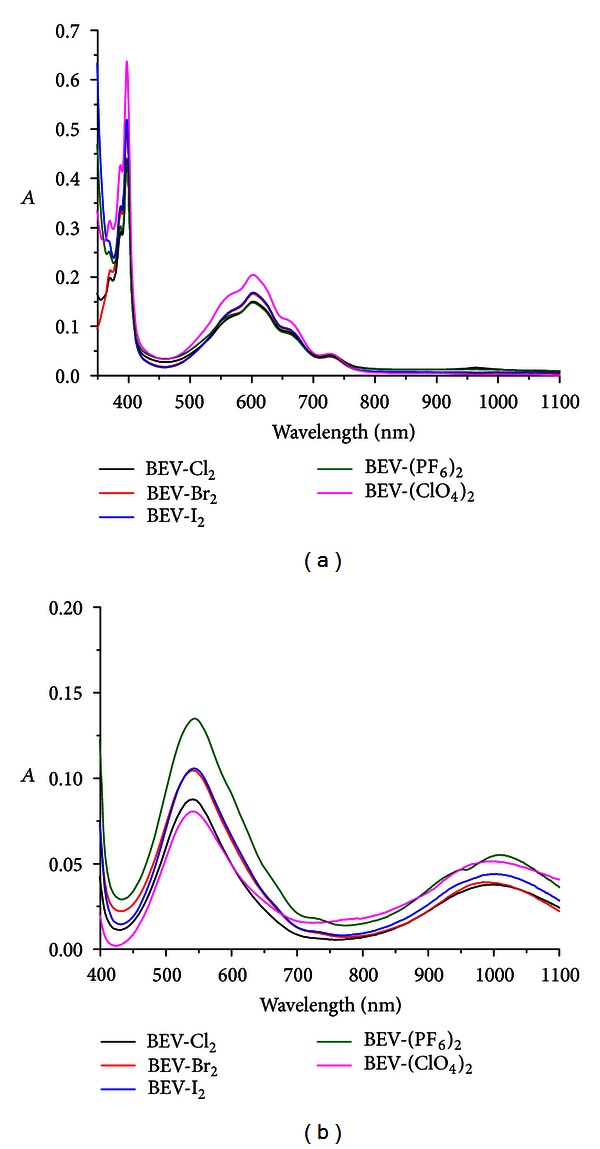
The absorption spectra of BEV-X_2_ (1 × 10^−5^ M) after reduction with Na_2_S_2_O_4_ (8–10 eq) under Ar atmosphere, (a) without CB[8], and (b) with the presence of 1 equivalent CB[8].

To provide additional information, UV-Vis spectra were taken after the BEV-X_2_ solution was reduced with excess Na_2_S_2_O_4_. All experiments were completed under an argon atmosphere and the results are listed in Figure 5.

After addition of excess Na_2_S_2_O_4_ (8–10 eq) into 10 *μ*M BEV-X_2_ in aqueous solution, the color of the solution turned to violet from colorless, and characteristic absorption [2, 26] of the viologen radical was observed at 400 nm and 600 nm for all the complexes. All these provided some further evidence that the counter anions were located outside the CB[8] cavity, while the different absorptivity that resulted demonstrated that the counter anions had some effect on the radical formation process. With the presence of CB[8], the color of the solution was changed to magenta after addition of excess Na_2_S_2_O_4_ (8–10 equivalent), and the characteristic absorptions for the radical dimer at 370 nm, 540 nm, and 1000 nm were observed simultaneously, illustrating the formation of the radical dimer in CB[8] cavity; all these are in good agreement with the literature [2, 26]. These quite different absorption profiles provide further evidence that the counter anions were located outside the CB[8] cavity, influencing the radical dimerization process too. 

 A control experiment was performed on Na_2_S_2_O_4_ (8–10 eq) solution without addition of any complexes (see Supplementary Figure S8). No signals can be followed in the corresponding UV-Vis spectral domain, providing further evidence that these results are caused by the anions present.

## 4. Conclusions

Viologen derivatives with different counter anions were successfully synthesized. NMR, MS, electrochemistry, and UV-Vis were employed to study the assembly of the target complexes with CB[8]. All results suggested that viologen region was threaded through the cavity of CB[8] while the counter anions were located outside the cavity. The counter anions do have some effect on Na_2_S_2_O_4_-induced viologen radical and its dimerization process. These data provide new insights into CB[8] host-guest chemistry. 

## Supplementary Material

NMR spectra of BEV-Cl2, BEV-Br2 and BEV-I2, ES-MS spectra, optimized geometries, electrochemistry and photophysical spectra of the inclusion complexes.

## Figures and Tables

**Table 1:** The key optimized atom distances and angles for the including complexes in the ground states, calculated with DFT at the B3LYP/3-21G level using Gaussian 09.

	BEV-Cl_2_	BEV-Br_2_	BEV-I_2_	BEV-(ClO_4_)_2_	BEV-(PF_6_)_2_
C_146_-N_152_ distance (angstrom)	5.008	5.024	5.003	5.025	5.034
A(C_149_-C_151_-N_152_) (degree)	109.68	110.04	109.55	110.92	111.13
D(N_152_-C_153_-C_154_-C_155_) (degree)	1.012	0.857	1.165	0.468	0.781
D(C_158_-C_159_-C_160_-N_161_) (degree)	0.235	0.392	−0.013	0.690	0.88
D(C_159_-C_160_-N_161_-C_162_) (degree)	2.136	1.893	2.178	2.166	1.883
D(C_146_-C_145_-C_150_-C_149_) (degree)	−0.922	−0.948	−1.054	−0.704	−0.926
D(C_150_-C_149_-C_151_-H_172_) (degree)	3.272	−2.983	0.603	11.387	9.167
