# Supplementary material for: Study of the Counter Anions in the Host-Guest Chemistry of Cucurbit[8]uril and 1-Ethyl-1′-benzyl-4,4′-bipyridinium
Source: ScientificWorldJournal. 2013 May 27;2013:452056. doi: 10.1155/2013/452056 (PMC3678417; doi:10.1155/2013/452056)
Supplement: Supplementary file 1 — NMR spectra of BEV-Cl2, BEV-Br2 and BEV-I2, ES-MS spectra, optimized geometries, electrochemistry and photophysical spectra of the inclusion complexes. [file 452056.f1.doc]

Supporting information


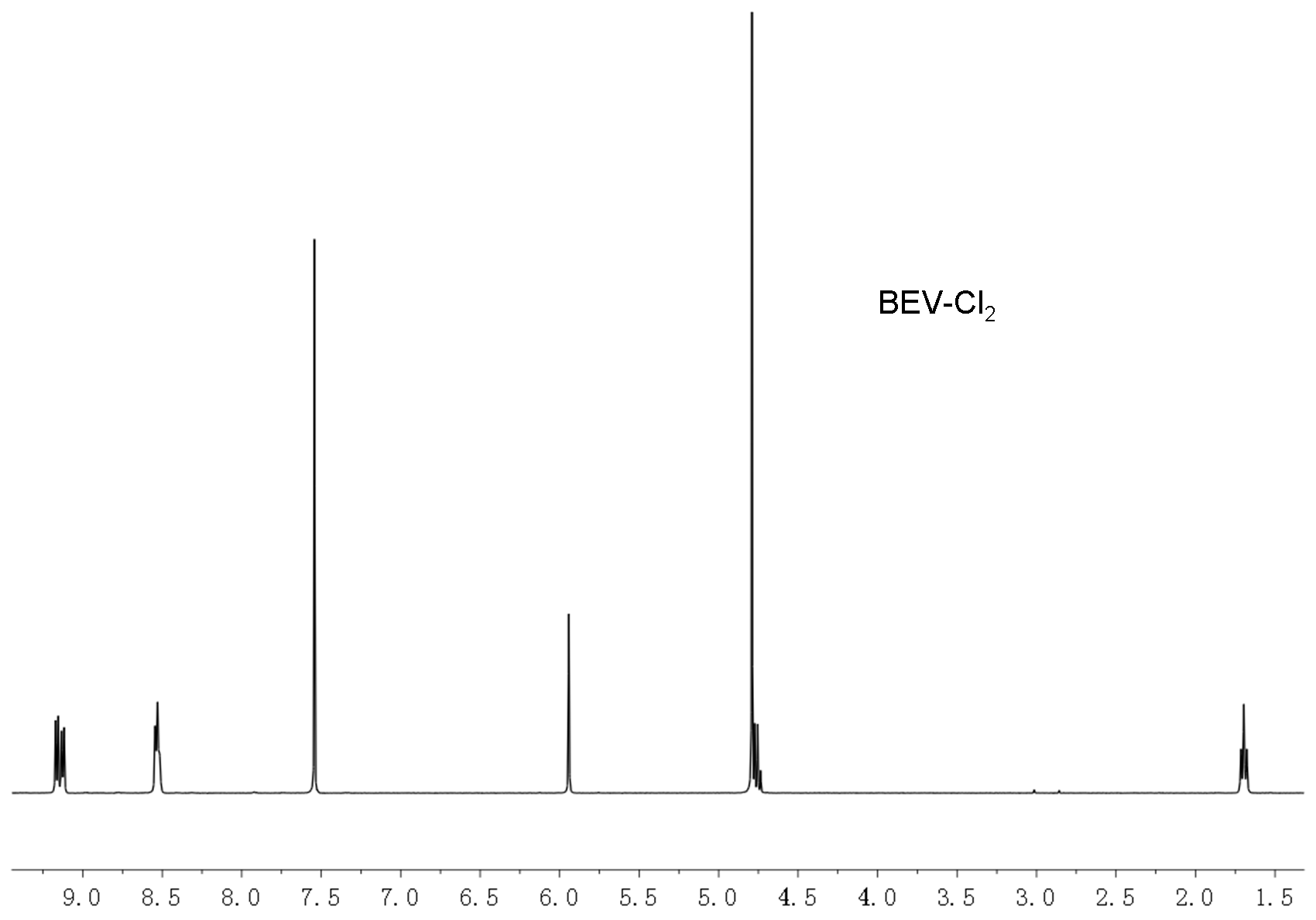


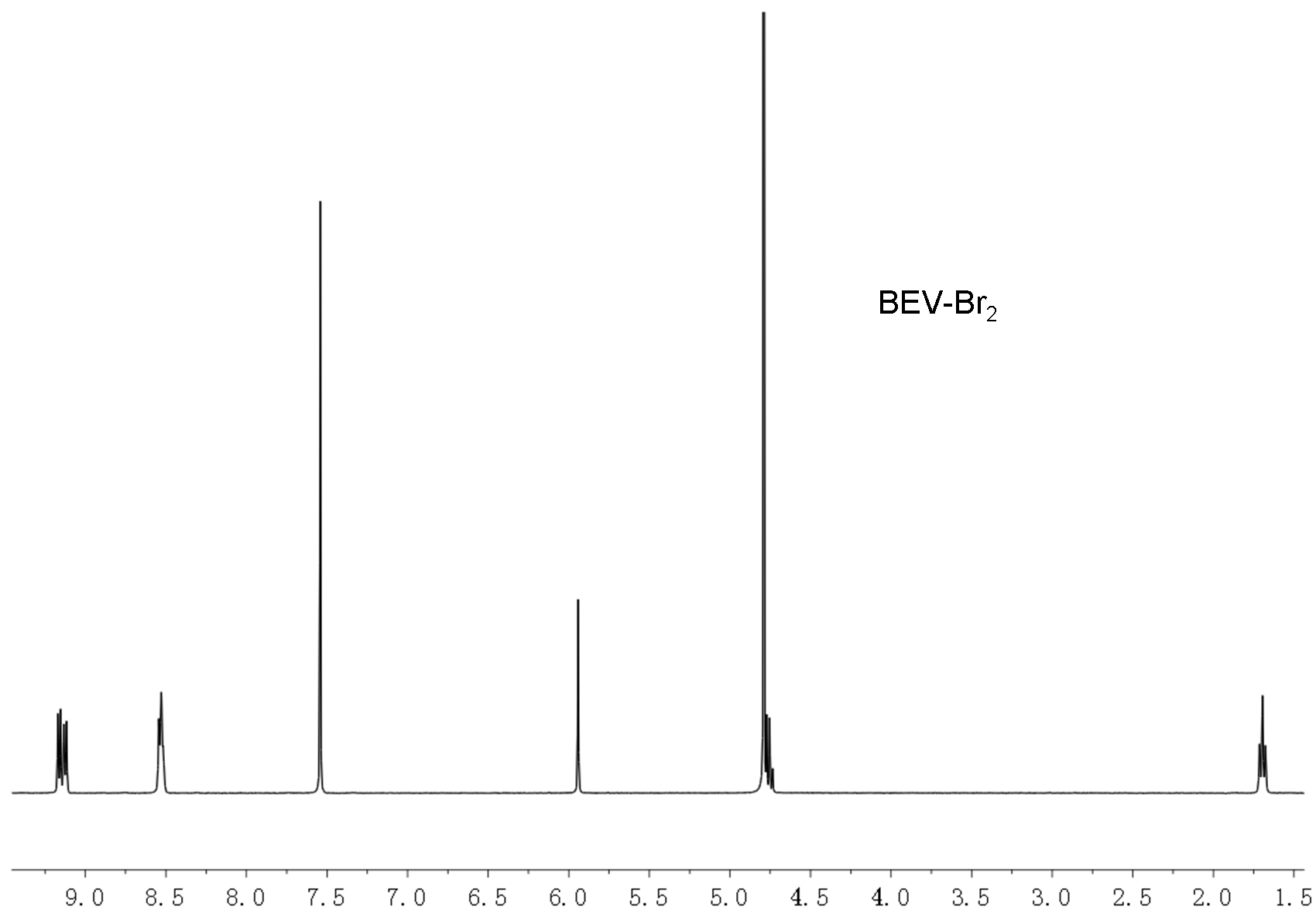


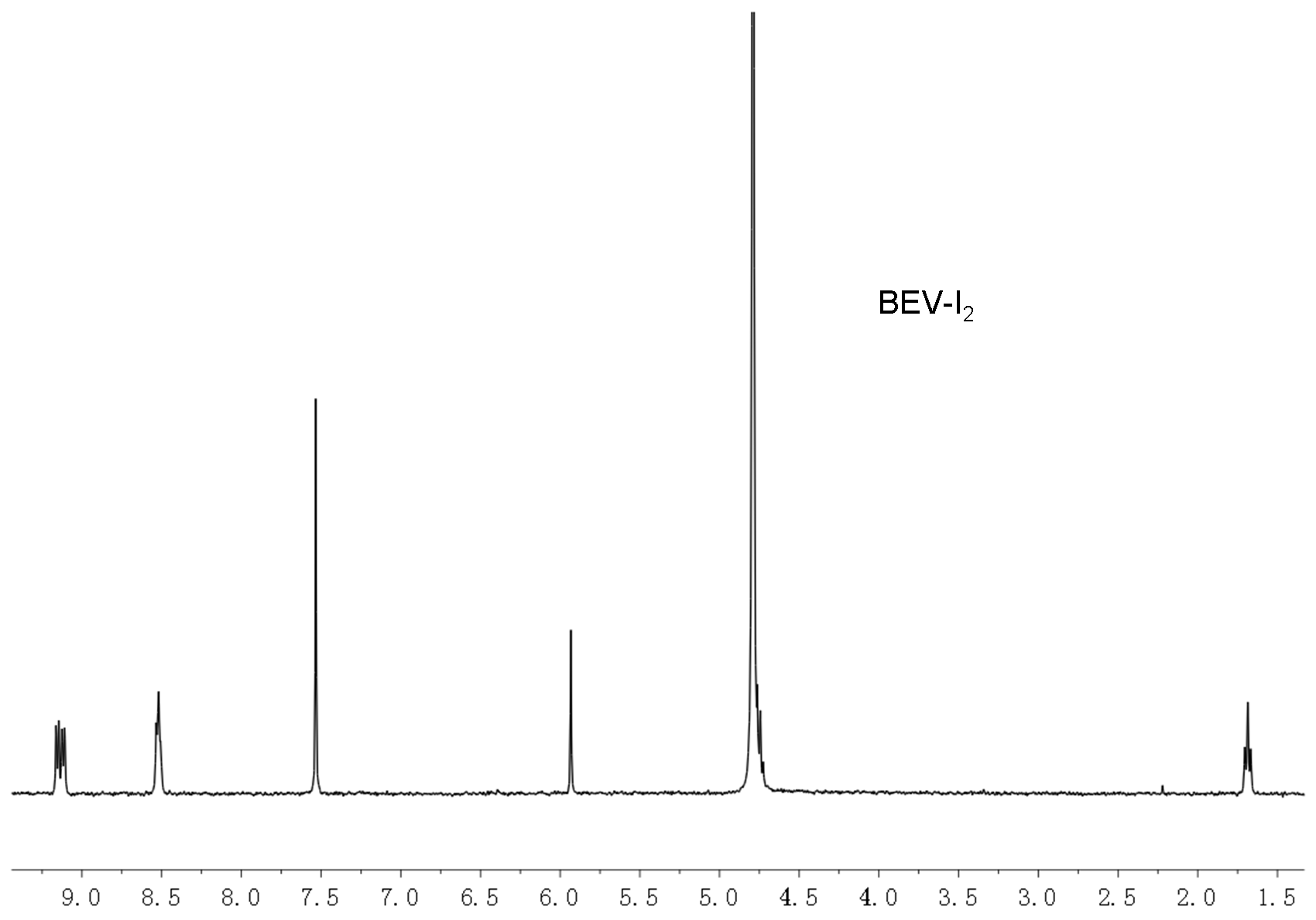


Fig. S1 ^1^H NMR spectra of BEV-Cl_2_, BEV-Br_2_ and BEV-I_2_ in D_2_O (400 MHz)

Fig. S2 ES-MS spectra of the 1:1 host-guest complexes BEV^2+^/CB[8], from up to down, the counter anion is Cl, Br, I, respectively.


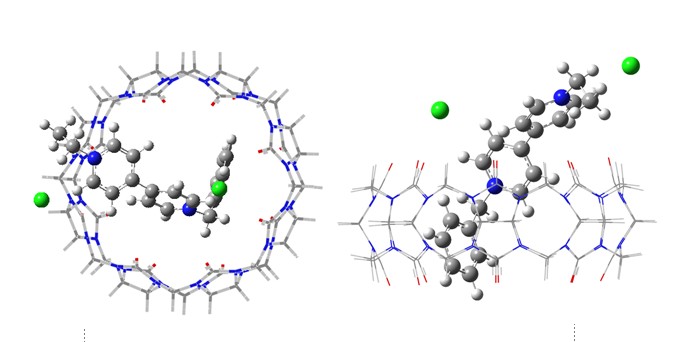

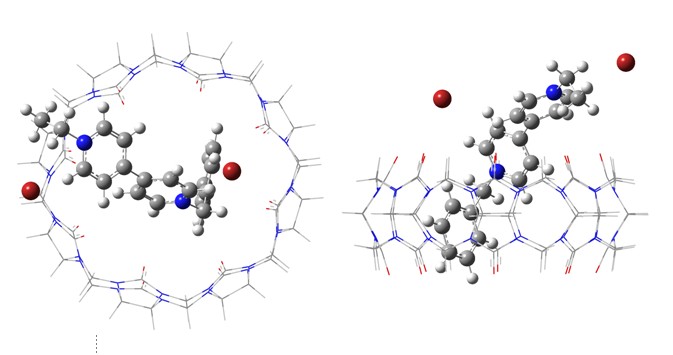

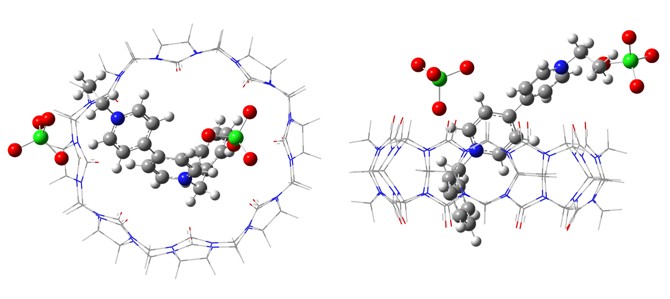

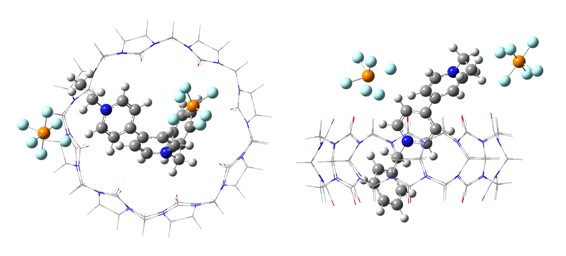


Fig. S3 The optimized geometries of the inclusion complexes BEV-X_2_/CB[8] viewed from front (left) and side (right). To aid visualization, CB[8] is in stick representation and the viologen salt in ball and stick representation with the X atoms in colour. From up to down, BEV-Cl_2_, BEV-Br_2_, BEV-(ClO_4_)_2_, and BEV-(PF_6_)_2_ respectively.

Fig. S4 CV (left) and DPV (right) curves for the 1:1 host-guest complexes BEV^2+^/CB[8] in 0.1 M PBS, from up to down, the counter anion is Cl, Br, I, PF_6_ and ClO_4_ respectively. The concentration of BEV-X_2_ was 0.5 mM.

Fig. S5 CV (left) and DPV (right) curves for 0.1 M PBS buffer alone.

Fig. S6 Control experiment on the UV-Vis titration of 1x10^-5^ M KI with 1.5 eq CB[8].

Fig. S7 Absorption responses of 10 μM BEV-X_2_ to CB[8] solutions for K_d_ value determination. From (A) to (E), the counter ion is Cl, Br, I, PF_6_ and ClO_4_, respectively. The concentration of BEV-X_2_ was 1x10^-5^ M.

Fig. S8 Control experiment on the UV-Vis of Na_2_S_2_O_4_ (8-10 eq) in aqueous solution
